# Association of Tagging SNPs in the *MTHFR* Gene with Risk of Type 2 Diabetes Mellitus and Serum Homocysteine Levels in a Chinese Population

**DOI:** 10.1155/2014/725731

**Published:** 2014-08-06

**Authors:** Han Wang, Cong Hu, Shu-Hui Xiao, Bin Wan

**Affiliations:** ^1^Undergraduate Student Brigade 11 Battalion, Third Military Medical University, Chongqing 400038, China; ^2^Center for Reproductive Medicine, The First Affiliated Hospital of Jilin University, Changchun 130062, China; ^3^Laboratory of Immune, People's Hospital of Linyi, No. 27 Eastern Jiefang Road, Lanshan, Linyi 276003, China

## Abstract

Diabetes is a global public health crisis, and the prevalence is increasing rapidly. Folate supplementation is proved to be effective in reducing the risk of diabetes or improving its symptoms. Methylenetetrahydrofolate reductase is an important enzyme involved in folate metabolism. The aim of this study is to examine whether polymorphisms in the *MTHFR* gene are associated with risk of type 2 diabetes mellitus (T2DM) and fasting total serum homocysteine (tHcy) levels. We genotyped nine tagging SNPs in the *MTHFR* gene in a case-control study, including 595 T2DM cases and 681 healthy controls in China. We found that C allele of rs9651118 had significant decreased risk of T2DM (adjusted odds ratio (OR) = 0.69, 95% confidence interval (CI): 0.55–0.87, *P* = 0.002) compared with T allele. Haplotype analysis also showed that *MTHFR* CTCCGA haplotype (rs12121543-rs13306553-rs9651118-rs1801133-rs2274976-rs1801131) had significant reduced risk of T2DM (adjusted OR = 0.71, 95% CI: 0.58–0.87, *P* = 0.001) compared with CTTTGA haplotype. Besides, the *MTHFR* rs1801133 was significantly associated with serum levels of tHcy in healthy controls (*P* = 0.0002). These associations were still significant after Bonferroni corrections (*P* < 0.0056). These findings suggest that variants in the *MTHFR* gene may influence the risk of T2DM and tHcy levels.

## 1. Introduction 

Diabetes mellitus (DM) is one of the primary chronic diseases that causes premature death and disability. It was estimated to be the third most challenging disease that is threatening public health just after malignant tumors and cardiocerebral vascular diseases in the world [1]. In recent years, the prevalence of DM was rising rapidly worldwide, particularly in developing countries [2]. In China, age-standardized incidence rate of DM was 2.4% in 1994 but raised to 9.7% in 2007-2008 [3, 4]. The importance of conducting prevention and early detection of DM has been increasingly emphasized.

Type 2 DM (T2DM) is the most common form of diabetes, which accounts for about 90% of all diagnosed DM cases. It is a multifactorial disease that is due to a combination of environmental and genetic risk factors, including dietary habit, obesity, physical inactivity, smoking, alcohol consumption, and genetic variants [5]. Studies have confirmed the relationship between homocysteinemia and diabetic complications, such as diabetic nephropathy, cardiovascular disease, and hypertension [6–8]. It is also reported that a high concentration of homocysteine is associated with the development of T2DM [9, 10]. MTHFR (methylenetetrahydrofolate reductase), a folate-dependent enzyme, plays an important role in the conversion of homocysteine to methionine by converting 5,10-methylenetetrahydrofolate to 5-methyltetrahydrofolate. Studies have found that specific genetic polymorphisms in the* MTHFR* gene could lead to change of MTHFR enzyme activity [11, 12]. Previous epidemiologic studies have also shown that genetic polymorphisms in* MTHFR* gene may be related to cancers, ischemic stroke, hypertension, and so forth [13–15]. But the association of* MTHFR* genotypes and susceptibility to T2DM in Chinese population has not been fully studied.

To help clarify whether the* MTHFR* variants are associated with susceptibility of T2DM and serum total homocysteine (tHcy) level, we examined nine tagging SNPs in the* MTHFR* gene (rs12121543, rs13306561, rs13306553, rs9651118, rs1801133, rs2274976, rs4846048, rs1801131, and rs17037396) in a case-control study in China.

## 2. Material and Methods

### 2.1. Study Participants

In this study, we consecutively enrolled 595 T2DM patients in the first affiliated hospital of Jilin university, Changchun, China, aged from 35 to 80, between June 2011 and June 2013. A case of T2DM was considered to be confirmed if the participant's reported glucose level met the well-established diagnostic criteria recommended by American diabetes association: with either the fasting plasma glucose (FPG) of ≥7.0 mmol/L or the 2-h value in the 75-g oral glucose tolerance test (OGTT) ≥11.1 mmol/L [16]. A total of 655 healthy control subjects were selected during the same period and from the same hospital and were frequency matched to the cases by age (5-year age groups) and gender. All controls were individuals free of diabetes that are determined by medical history or clinical examinations. At enrollment, demographic and medical histories, including age, gender, body mass index, smoking habits, and hypertension status were collected from each subject by a trained interviewer using a structured questionnaire. Blood samples were collected after a 12-hour overnight fast and then separated into serum, red blood cells, and buffy coat. Written informed consent was obtained from all enrolled participants and this study was approved by the Ethics Committee of Jilin University.

### 2.2. SNP Selection

Tag SNPs were selected by searching Han Chinese data from the HapMap project (http://www.hapmap.org/) using the Tagger program. The following criteria were used to identify tagging SNPs: (a) SNPs located in the gene or within the 2-kb region flanking the gene, (b) a minor allele frequency ≥0.1, and (c) other unselected SNPs could be captured by one of the tagging SNPs with a linkage disequilibrium of *r* ^2^ ≥ 0.90. As a result, a total of 9 tag SNPs were identified.

### 2.3. Laboratory Tests

Serum levels of total cholesterol (TC), low density lipoprotein-cholesterol (LDL-C), and high density lipoprotein-cholesterol (HDL-C) were determined in cases and controls by commercial kits from the Nanjing Jiancheng Bio-company (Nanjing, China). Serum tHcy levels in healthy controls were measured by cycling enzymatic method (Nanjing Jiancheng Bio-company, Nanjing, China) and were analyzed with an automatic biochemical analyzer (AU 2700 Olympus, First Chemical Ltd, Tokyo, Japan).

Genomic DNA was extracted from peripheral blood leucocytes using DNA Extraction Kit (Qiagen, Hilden, Germany). Genotyping was performed on the ABI PRISM 7900HT Sequence Detection System (Applied Biosystems, Foster City, CA, USA), using the TaqMan assay. The genotyping call rate was >95%, and the completion rate was >99%. The quality and potential misclassification of the genotyping were assessed by regenotyping 10% of duplicate DNA samples that were randomly selected from the whole population and placed within the same reaction plates used for the study subjects. The concordance rate for the quality control samples was 100%.

### 2.4. Statistical Analysis

We used SAS software (version 9.3; SAS Institute, Inc.) for the statistical analyses. *χ* ^2^ statistics and the *t*-test were used to evaluate case-control differences in the distribution of risk factors. Variables were tested for normality with Shapiro-Wilk statistics. Skewed data, including age, BMI, TC, LDL-C, HDL-C, and tHcy, were log transformed and expressed as medians and interquartile ranges. A two tailed *P* value of 0.05 was considered statistically significant. The odds ratios (ORs) and 95% confidence intervals (CIs) for the associations between the SNPs and T2DM risk were estimated by unconditional logistic regression. Hardy-Weinberg equilibrium for genotypic distribution and linkage disequilibrium between loci were assessed by HaploView version 4.0 (Daly Lab at the Broad Institute, Cambridge, MA, USA) [17]. Associations between haplotypes (>1% frequency) and the risk of T2DM were evaluated by computing OR and 95% CI using HAPSTAT, assuming an additive model, using the most common haplotype as the referent category [18]. Both univariate ANOVA and multivariate ANCOVA analyses adjusting for age, sex, BMI, and hypertension were performed to determine the effects of the* MTHFR* polymorphisms on serum tHcy levels in healthy controls. *P* values (from the additive model or dominant model) of less than 0.0056 were deemed to be statistically significant after Bonferroni corrections.

## 3. Results

Characteristics of the study subjects are shown in Table 1. Cases and controls were evenly matched by age and gender. Cases were more probably to have higher BMI (24.6 kg/m^2^ versus 23.1 kg/m^2^) and hypertension (43.9% versus 24.5%). Besides, cases have significant lower levels of serum HDL-C and higher levels of serum total cholesterol and LDL-C than that in controls.

The associations of* MTHFR* variants and risk of T2DM are presented in Table 2. The genotype distributions of these nine polymorphisms showed no deviation from the expected Hardy-Weinberg equilibrium among controls (*P* > 0.05). Of these SNPs, C allele of rs9651118 conferred significant lowered risk of T2DM (crude OR = 0.71, 95% CI: 0.57–0.88, *P* = 0.002) compared with T allele, and rs1801131 AC genotype conferred significant increased risk of T2DM compared with AA genotype (crude OR = 1.29, 95% CI: 1.02–1.64, *P* = 0.03). These associations remained significant after adjustment for other risk factors (age, sex, BMI, and hypertension). But only rs9651118 was still significant after Bonferroni correction in dominant model (adjusted OR = 0.69, 95% CI: 0.55–0.87, *P* = 0.002). None of the other SNPs examined was associated with the risk of T2DM.

Six SNPs in the* MTHFR* gene (rs12121543, rs13306553, rs9651118, rs1801133, rs2274976, and rs1801131) were in linkage disequilibrium with D′ ranging from 0.79 to 0.99 and *r* ^2^ ranging from 0.04 to 0.86. Subjects carrying the* MTHFR* CTCCGA haplotype had a significant reduced risk of T2DM (OR = 0.73, 95% CI: 0.60–0.88, *P* = 0.001) compared with those carrying the CTTTGA haplotype (Table 3). This association remained significant after adjustment for other risk factors and after Bonferroni correction.

Finally, we investigated the associations between the* MTHFR* SNPs and serum tHcy levels in the control population. Carriers of the mutant alleles of rs1801133 were significantly associated with increased serum level of tHcy [C/T: 12.8(10.9–14.4) *μ*mol/L; T/T: 11.9(9.5–16.6) *μ*mol/L] compared with carriers of the CC genotype [11.3(9.3–13.7) *μ*mol/L]. Rs9651118 T/C and C/C genotypes [12.1(10.1–14.1) *μ*mol/L; 11.3(9.3–13.4) *μ*mol/L] conferred significant decreased serum level of tHcy in controls compared with TT genotype [12.5(9.8–14.6) *μ*mol/L]. These associations were also significant in multivariate ANCOVA analyses. But after Bonferroni correction only rs1801133 was significantly associated with serum level of tHcy. None of the other studied SNPs was associated with serum tHcy level (Table 4).

## 4. Discussion

In this molecular epidemiologic study, polymorphisms in the* MTHFR* gene were fully studied for their association with susceptibility to T2DM and serum tHcy levels. We demonstrated that one genetic mutation (rs9651118) in* MTHFR* gene was significantly associated with risk of T2DM, and rs1801133 was significantly associated with serum tHcy levels in healthy population.

*MTHFR* gene is located on chromosome 1p36.3 in humans. To date, over 40 point genetic mutations in* MTHFR* gene have been identified, of which A1298C (rs1801131) and C677T (rs1801133) showed the most clinical significance. The* MTHFR* rs1801133 is a missense mutation that results in approximately 70% and 35% reduction of normal MTHFR enzyme activity in TT and CT genotype carriers, respectively [11]. MTHFR activity has an adverse effect on serum tHcy level.* MTHFR* rs1801133 TT genotype was also associated with elevated tHcy levels, predominantly in individuals who have a low plasma folate level (<15.4 nmol/L), compared to the normal genotype [19]. In our study, although T allele of rs1801133 has nonsignificant raised risk of T2DM (OR = 1.22, 95%CI: 0.97–1.55), we found that the mutant alleles were associated with significantly higher level of tHcy in healthy controls. Besides, we observed that the average tHcy level in TT genotype carriers was 13.2 *μ*mol/L which was higher than that in CT genotype carriers (13.0 *μ*mol/L). These results were in accordance with previous studies.

A1298C (rs1801131) is a common mutation in the* MTHFR* gene that results in the conversion of adenine to cytosine, which results in reduction of MTHFR enzyme activity [20]. Few studies have evaluated the relationship between the* MTHFR* A1298C polymorphism and susceptibility of diabetes, and the results remain inclusive. Previous study suggested that* MTHFR* A1298C polymorphism is a risk factor for T2DM in Egyptian patients, while other failed to find any association in Taiwanese or Moroccan [21–23]. A recent Meta-analysis found that* MTHFR* A1298C has a significant association with diabetes in Asian population under dominant model but not in Caucasians [24]. The association between A1298C and serum homocysteine level was also controversial. Kumar et al. found that subjects with* MTHFR* rs1801131 CC genotype had significant higher level of homocysteine (16.3 *μ*mol/L) compared with AA genotype (14.4 *μ*mol/L) in Indian population, but this association was not found in Scotch [25, 26]. In our study, although we found AC genotype had relatively higher risk of T2DM, the significance no longer exists after Bonferroni correction. In addition, we did not find its relation with homocysteine level.

Rs9651118 is located in the intron region in* MTHFR* gene with unknown genetic function. It was once reported that C allele of rs9651118 was associated with reduced lung cancer risk in never smokers, and a nominally significant association with schizophrenia in the form of haplotypes (rs1801133, rs17421511, rs17037396, and rs9651118) in Japanese population [27, 28]. Rs9651118 TT genotype was reported to confer significant elevated tHcy level compared with CC genotype in a population-based CoLaus study [29]. In our research, rs9651118 was associated with risk of T2DM, and we also found that carriers of TT genotype had nonsignificant higher level of tHcy relative to CC genotype carriers, which was in accordance with the previous study.

## 5. Conclusion

In conclusion, the present study suggests that* MTHFR* rs9651118 was associated with T2DM risk and* MTHFR* rs1801133 may affect serum tHcy levels.

## Figures and Tables

**Table 1:** Selected characteristics of cases and controls.

Characteristics	Cases (*n* = 595)	Controls (*n* = 681)	*P* value
Age (year)	65 (56–71)	64 (56–73)	0.43
Sex (male/female)	321/274	375/306	0.69
BMI (kg/m^2^)	24.6 (22.7–27.0)	23.1 (21.1–25.4)	<0.001
Smoking (yes/no)	182/413	229/452	0.25
Hypertension (yes/no)	261/334	167/514	<0.001
Total cholesterol (mmol/L)	4.68 (4.09–5.48)	4.14 (3.60–5.07)	<0.001
HDL-C (mmol/L)	1.10 (0.98–1.42)	1.24 (1.09–1.58)	<0.001
LDL-C (mmol/L)	2.81 (2.45–3.29)	2.38 (2.05–2.93)	<0.001

**Table 2:** ORs and 95% CIs for T2DM in relation to polymorphisms of *MTHFR* gene.

SNP	Genotypes	Cases, *n* (%)	Controls, *n* (%)	OR (95% CI)∗	OR (95% CI)^†^	*P* trend^‡^
rs12121543	CC	402 (67.7)	462 (67.9)	1.00	1.00	0.79
	CA	177 (29.8)	194 (28.5)	1.04 (0.82–1.33)	1.07 (0.83–1.38)	
	AA	15 (2.5)	24 (3.5)	0.71 (0.37–1.37)	0.77 (0.38–1.54)	
	CA + AA	192 (32.3)	218 (32.1)	1.01 (0.80–1.27)	1.04 (0.81–1.33)	

rs13306561	TT	474 (79.8)	543 (79.8)	1.00	1.00	0.98
	TC	116 (19.5)	131 (19.3)	1.01 (0.76–1.33)	1.04 (0.77–1.39)	
	CC	4 (0.7)	6 (0.9)	0.73 (0.20–2.61)	0.58 (0.15–2.21)	
	TC + CC	120 (20.2)	137 (20.1)	1.00 (0.76–1.31)	1.01 (0.76–1.35)	

rs13306553	TT	480 (80.8)	547 (80.4)	1.00	1.00	0.82
	TC	110 (18.5)	129 (19.0)	0.97 (0.73–1.28)	0.97 (0.72–1.30)	
	CC	4 (0.7)	4 (0.6)	1.08 (0.27–4.36)	1.06 (0.25–4.50)	
	TC + CC	114 (19.2)	133 (19.6)	0.97 (0.73–1.28)	0.97 (0.73–1.30)	

rs9651118	TT	303 (51.0)	288 (42.4)	1.00	1.00	0.009
	TC	231 (38.9)	300 (44.1)	0.73 (0.58–0.93)	0.72 (0.56–0.93)	
	CC	60 (10.1)	92 (13.5)	0.62 (0.43–0.90)	0.59 (0.40–0.87)	
	TC + CC	291 (49.0)	392 (57.6)	0.71 (0.57–0.88)	0.69 (0.55–0.87)	

rs1801133	CC	234 (39.5)	298 (43.8)	1.00	1.00	0.14
	CT	293 (49.4)	312 (45.9)	1.20 (0.95–1.51)	1.22 (0.96–1.56)	
	TT	66 (11.1)	70 (10.3)	1.22 (0.83–1.77)	1.23 (0.83–1.84)	
	CT + TT	359 (60.5)	382 (56.2)	1.20 (0.96–1.50)	1.22 (0.97–1.55)	

rs2274976	GG	484 (81.5)	554 (81.5)	1.00	1.00	0.99
	GA	105 (17.7)	119 (17.5)	1.00 (0.75–1.34)	1.02 (0.75–1.38)	
	AA	5 (0.8)	7 (1.0)	0.78 (0.25–2.49)	0.62 (0.19–2.04)	
	GA + AA	110 (18.5)	126 (18.5)	0.99 (0.75–1.32)	1.00 (0.74–1.34)	

rs4846048	AA	490 (82.5)	560 (82.3)	1.00	1.00	0.95
	AG	99 (16.7)	114 (16.8)	0.99 (0.74–1.33)	1.01 (0.74–1.38)	
	GG	5 (0.8)	6 (0.9)	0.97 (0.29–3.19)	1.39 (0.40–4.88)	
	AG + GG	104 (17.5)	120 (17.6)	0.99 (0.74–1.32)	1.03 (0.76–1.39)	

rs1801131	AA	370 (62.3)	455 (66.9)	1.00	1.00	0.04
	AC	208 (35.0)	197 (29.0)	1.29 (1.02–1.64)	1.32 (1.03–1.70)	
	CC	16 (2.7)	28 (4.1)	0.69 (0.37–1.30)	0.74 (0.38–1.44)	
	AC + CC	224 (37.7)	225 (33.1)	1.22 (0.97–1.54)	1.25 (0.98–1.59)	

rs17037396	CC	456 (76.8)	521 (76.5)	1.00	1.00	0.88
	CT	126 (21.2)	147 (21.6)	0.98 (0.75–1.28)	1.03 (0.78–1.37)	
	TT	12 (2.0)	13 (1.9)	1.07 (0.48–2.36)	0.93 (0.41–2.11)	
	CT + TT	138 (23.2)	160 (23.5)	0.98 (0.76–1.28)	1.02 (0.78–1.34)	

*Adjusted for age. ^†^Adjusted for age, sex, BMI, and hypertension.

^‡^Test of trend for the number of copies of the variant allele (0, 1, and 2).

**Table 3:** Association of haplotypes in the *MTHFR * gene with risk of T2DM.

Haplotype∗	Cases, %	Controls, %	OR^†^ (95% CI)	OR (95% CI)^‡^
CTTTGA	34.9	32.2	1.00	1.00
CTCCGA	26.9	35.3	0.73 (0.60–0.88)	0.71 (0.58–0.87)
ATTCGC	17.3	12.5	1.22 (0.95–1.56)	1.20 (0.92–1.55)
ACTCAC	8.6	8.2	0.92 (0.67–1.25)	0.93 (0.67–1.29)
CTTCGA	6.7	8.8	0.79 (0.57–1.10)	0.80 (0.56–1.13)

*In the order rs12121543, rs13306553, rs9651118, rs1801133, rs2274976, and rs1801131.

^†^Adjusted for age. ^‡^Adjusted for age, sex, BMI, and hypertension.

**Table 4:** Association between *MTHFR* polymorphisms and serum tHcy levels in health controls.

SNP	M/m	tHcy (*μ*mol/L)			P*
		MM	Mm	mm	
rs12121543	C/A	12.5 (10.1–14.6)	11.8 (9.7–13.5)	10.4 (8.5–14.1)	0.21
rs13306561	T/C	12.1 (9.9–14.4)	10.5 (8.4–13.7)	12.3 (10.0–14.4)	0.87
rs13306553	T/C	12.1 (10.0–14.4)	12.4 (10.0–14.4)	9.1 (8.1–11.8)	0.45
rs9651118	T/C	12.5 (9.8–14.6)	12.1 (10.1–14.1)	11.3 (9.3–13.4)	0.02
rs1801133	C/T	11.3 (9.3–13.7)	12.8 (10.9–14.4)	11.9 (9.5–16.6)	0.0002
rs2274976	G/A	12.1 (9.9–14.4)	12.3 (10.0–14.1)	10.7 (8.4–13.7)	0.84
rs4846048	A/G	12.4 (10.0–14.4)	11.4 (9.5–13.3)	10.7 (8.6–20.1)	0.28
rs1801131	A/C	12.5 (10.1–14.6)	11.8 (9.7–13.6)	10.8 (8.6–14.1)	0.26
rs17037396	C/T	12.1 (9.9–14.2)	12.1 (10.0–14.6)	13.7 (12.5–15.2)	0.67

*Adjusted for age, sex, BMI, and hypertension.

M indicates major allele; m indicates minor allele.
